# Complete chloroplast genome of a cultivated oil camellia species, *Camellia gigantocarpa*

**DOI:** 10.1080/23802359.2021.2008836

**Published:** 2021-12-10

**Authors:** Yufen Xu, Yanju Liu, Xiaocheng Jia

**Affiliations:** Hainan Key Laboratory of Tropical Oil Crops Biology/Coconut Research Institute, Chinese Academy of Tropical Agricultural Sciences, Wenchang, China

**Keywords:** *Camellia*, Theaceae, complete chloroplast genome

## Abstract

*Camellia gigantocarpa* Hu et T. C. Huang, belonging to the Theaceae family, is an excellent landscape tree species with high ornamental value. It is also an important woody oil-bearing plant with high economic value. This study reports the first complete chloroplast genome sequence of *C. gigantocarpa* (GenBank accession number: MZ054232). Its whole chloroplast genome is 156,953 bp long with an overall GC content of 37.31%, which is composed of a large single copy region (86,631 bp), a small single copy region (18,402 bp), and a pair of inverted repeat regions (25,960 bp each). A total of 135 genes were predicted in this genome, namely eight ribosomal RNA genes, 37 transfer RNA genes, and 90 protein-coding genes. Based on maximum likelihood analysis results, we found that the *Camellia* species are clustered into a distinct branch, and the phylogenetic relationships among *C. gigantocarpa*, *C. crapnelliana*, and *C. kissii* were the closest.

In 1965, Hu Hsen-Hsu first published *Camellia gigantocarpa* Hu et T. C. Huang as a new species (Hu 1965). This species belongs to the Theaceae family, and is naturally distributed in the Bobai and Luchuan areas of Guangxi Province in China (Huang et al. 2021). *Camellia gigantocarpa* has a high ornamental value and is an excellent landscape tree species with a beautiful tree shape, large flowers, and large fruits. Moreover, seeds of this species have a high oil content and can be used for edible oil extraction with high economic value (Fan 2011). As an important woody oil-bearing plant, *C. gigantocarpa* is also one of the main cultivated species of oil-tea camellia in China (Dai et al. 2021). Research on *C. gigantocarpa* has mainly focused on genetic diversity (Fan et al. 2011; Peng et al. 2013), photosynthetic characteristics (Wu 2019), camellia seed oil composition, and soil heavy-metal remediation (Zhang et al. 2010); however, only a few studies on its taxonomy and phylogeny exist. In this report, we present the first complete chloroplast genome sequence of *C. gigantocarpa* and evaluate its phylogenetic relationships with related species.

Samples of *C. gigantocarpa* were collected from the Germplasm Repository of Oil Camellia at the Coconut Research Institute of the Chinese Academy of Tropical Agricultural Sciences (CATAS) (Hainan, China; coordinates: 19°32′4.80″ N, 110°45′47.43″ E). The sample (BB01) and voucher herbarium (BB1) were deposited in the Camellia Research Center of the Coconut Research Institute of CATAS. Total genomic DNA was extracted from the leaf material using a modified CTAB method (Doyle 1987). The library was constructed with an insert length of 350 bp and paired-end sequenced with 150-bp reads on the Illumina HiSeq2500 second-generation sequencing platform. In total, approximately 10.79 GB of raw reads was generated. With *C. pubicosta* (NC_024662.1) as the reference, the toolkit GetOrganelle (Jin et al. 2020) was used to assemble the chloroplast genome de novo. Similar to gene annotation, the starting positions of the chloroplast genome and the inverted repeat (IR) region were determined using the online annotation software Geseq (Tillich et al. 2017) (https://chlorobox.mpimp-golm.mpg.de/geseq.html) and CpGAVAS2 (Shi et al. 2019) (http://www.herbalgenomics.org/cpgavas). Finally, manual checking was performed to ensure that the annotations were correct, and the complete chloroplast genome of *C. gigantocarpa* was submitted to GenBank (accession number: MZ054232).

The entire chloroplast genome of *C. gigantocarpa* is a typical circular quadripartite structure 156,953 bp in length; the genome includes a large single copy region (LSC, 86,631 bp), a small single copy region (SSC, 18,402 bp), and a pair of IR regions (25,960 bp each). The overall GC content was 37.31%. According to the annotation results, the total number of functional genes was 135, consisting of eight ribosomal RNA genes, 37 transfer RNA genes, and 90 protein-coding genes. And the annotated gene names in chloroplast genome from *C*. *gigantocarpa* were listed in Supplemental material 1. Similar to most angiosperms, the chloroplast genome of *C*. *gigantocarpa* lost characteristic chloroplast genes of algae, bryophytes, pteridophytes, and gymnosperms, that is *psaM*, *psb30*, *chlB*, *chlL*, *chlN*, and *rpl21*. However, unlike most angiosperms, the chloroplast genome of *C. gigantocarpa* lost *atpI* and *rpl36* genes as well. This may be attributable to the reorganization of the chloroplast genome during the evolution of different lineages (Mohanta et al. 2020).

To explore the phylogenetic relationships within different *Camellia* species, a maximum likelihood phylogeny analysis was performed using IQ-TREE 2.1.3 (Minh et al. 2020). The best model for the complete chloroplast genomes of *C. gigantocarpa* (MZ054232), 21 other *Camellia* species and 4 outgroup species from Theaceae was K3Pu + F + I; 1,000 bootstrap replicates were used for this purpose. As illustrated in Figure 1, all the 22 *Camellia* species distinctly clustered into one large branch with relatively short internal evolutionary branches, whereas the other four species formed a separate branch with the relatively long internal evolutionary branches. This indicates that chloroplast genomes of *Camellia* species are distinctly and genetically different from those of the four outgroup Theaceae species, while the chloroplast genomes of *Camellia* species have a relatively high degree of genetic similarity. Furthermore, our results indicate that the *Camellia* genus is a monophyletic group, which is consistent with the findings of Yu et al. (2017). We also found close genetic relationships among *C. gigantocarpa*, *C. crapnelliana*, and *C. kissii*. We consider that the report provides a scientific basis for the molecular phylogeny and molecular breeding of *Camellia* species for research and industrial purposes.

**Figure 1.**
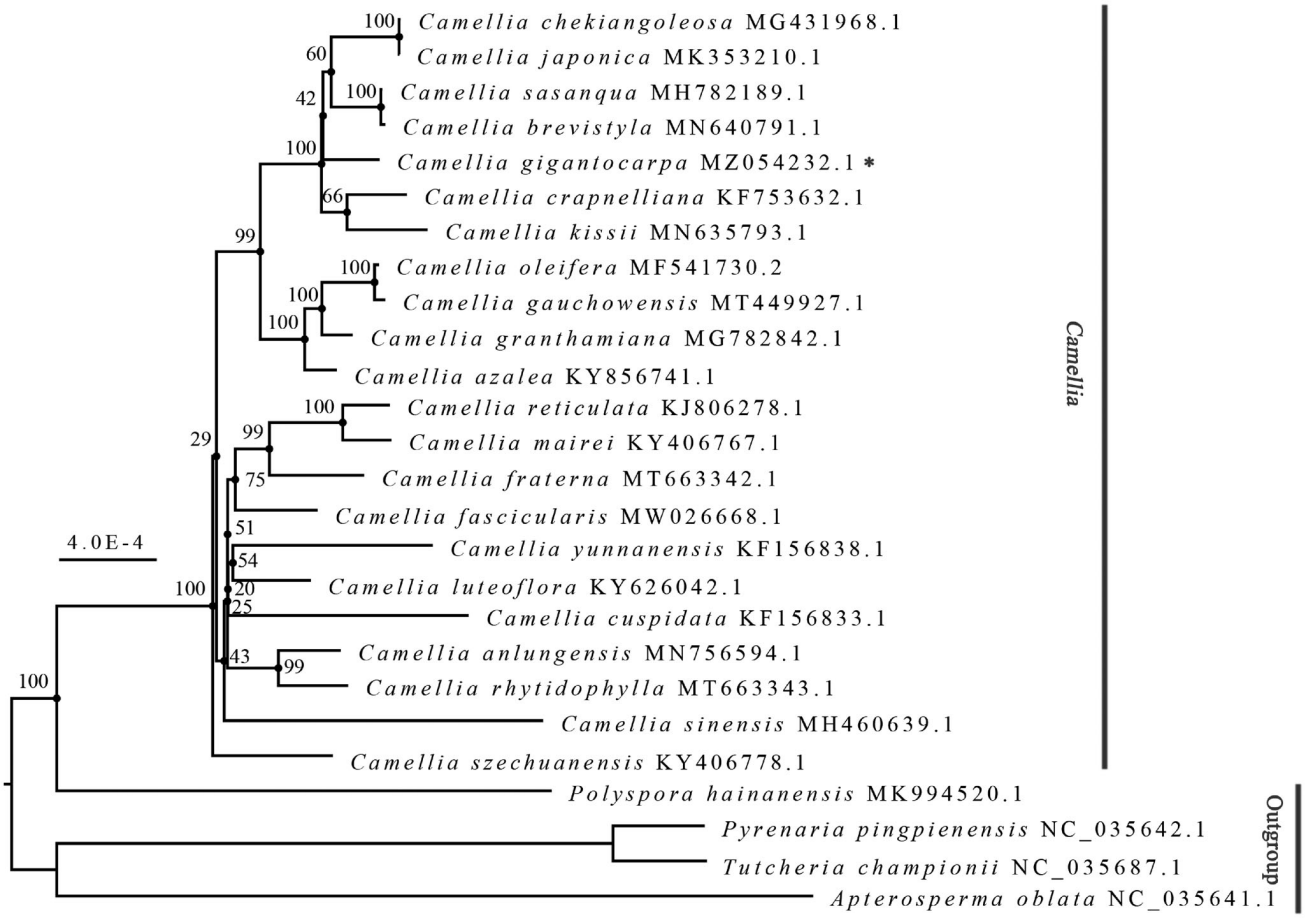
The maximum likelihood phylogenetic tree based on 26 chloroplast genome sequences of the Theaceae family. Bootstrap values based on 1,000 replicates are indicated at each branch node.

## Data Availability

The genome sequence data that support the findings of this study are openly available in GenBank of NCBI at (https://www.ncbi.nlm.nih.gov/) under the accession no. MZ054232. The associated BioProject, Bio-Sample, and SRA numbers are PRJNA725044, SAMN20667270, and SRR15371496, respectively.
